# Associations of Midlife Gait Speed and Muscle Performance With Falls in Later Life: Secondary Analysis of the MIDUS Cohort

**DOI:** 10.2196/103917

**Published:** 2026-10-06

**Authors:** Roee Hayek, Yaniv Nudelman, Dan Keren, Shmuel Springer

**Affiliations:** 1 Ariel University Ariel Israel

**Keywords:** middle age, mobility, fall risk, subjective age, physical performance

## Abstract

**Background:**

Falls are a major cause of morbidity among older adults, yet fall risk may begin to emerge during midlife. Physical performance measures may identify early functional vulnerability associated with later-life health outcomes.

**Objective:**

This study examined whether midlife gait speed and muscle performance were associated with recurrent falls later in life.

**Methods:**

This study was a secondary analysis of prospectively collected data from the Midlife in the United States (MIDUS) cohort. Independent adults (n=309) aged 45 to 64 years at wave 2 (2004-2009) who completed physical performance assessments were followed to wave 3 (2013-2021). Baseline measures included gait speed from a 50-foot walk test, grip strength, sit-to-stand time, and calculated lower-extremity power. The primary analysis examined associations between baseline physical performance and recurrent falls at follow-up (≥2 falls in the previous 12 months). A sensitivity analysis repeated the primary models but was restricted to participants without a baseline history of recurrent falls.

**Results:**

At follow-up, 20.4% (63/309) of participants reported recurrent falls. Slower midlife gait speed was associated with higher odds of recurrent falls (odds ratio 1.43, 95% CI 1.05-1.95; *P*=.02), whereas muscle performance measures were not associated with falls in the primary analysis. The association between slower gait speed and recurrent falls remained significant in the sensitivity analysis. Over follow-up, muscle performance declined, whereas gait speed remained stable.

**Conclusions:**

Slower midlife gait speed was associated with higher odds of recurrent falls, suggesting that fall vulnerability may be detectable as early as midlife. Given the exploratory nature of this study, further studies are warranted to confirm these findings.

## Introduction

Maintaining locomotor ability across the adult lifespan is a central goal of healthy aging. In many cases, increased life expectancy is not accompanied by preserved functional capacity, making mobility-related outcomes in later life a major health concern [1]. At the individual level, mobility limitations in older adulthood are associated with reduced participation, loss of independence, and increased fall risk [2]. Falls represent a substantial public health burden, contributing to substantial health care use, economic costs, and mortality among older adults [3].

Fall prevention efforts focus on identifying risk factors in clinical and research settings. Among these, markers of physical function are particularly informative because they reflect mobility capacity and physiological reserve and have been associated with increased fall risk and adverse health outcomes [4]. Recurrent falls, defined as 2 or more falls within 12 months, are of particular concern because they are more strongly associated with frailty, hospitalization, and mortality than isolated falls [3]. Despite extensive research and prevention initiatives, population-level progress remains limited, with increasing fall-related mortality among US adults aged 65 years and older [5,6], and annual fall prevalence remaining approximately 1 in 4 [6].

Importantly, falls are not limited to older adulthood, and midlife may represent a critical window for prevention. Recent harmonized cohort analyses across multiple countries report an annual fall prevalence of 17% to 28% among community-dwelling middle-aged adults. Fall rates increase markedly across midlife, nearly tripling from early (45-54 years) to late (55-64 years) midlife [7,8]. Furthermore, approximately 1 in 5 middle-aged adults report mobility limitations despite the absence of overt clinical conditions [9]. These impairments are often unrecognized in routine care, highlighting the importance of early detection of mobility decline to enable timely preventive interventions [8,10]. Screening physical performance during midlife may therefore provide insight into early functional vulnerability.

To better understand functional aging dynamics, several population-based cohorts initiate assessments in midlife to support a life-course perspective on aging [8,11-14]. One such example is the Midlife in the United States (MIDUS) study, a national longitudinal cohort that integrates objective assessments of physical function with psychosocial and health measures [12,13]. These include fall prevalence as well as self-rated health and subjective age, indicators that have been independently associated with subsequent physical decline, frailty, disability, and mortality [15-17]. Together, this multidimensional framework enables the examination of physical function alongside health perceptions in midlife and their relevance to aging-related outcomes.

Among the wide range of physical function variables available in MIDUS, gait speed and muscle performance capture distinct yet complementary aspects of physical function and mobility. These domains have been widely used to elucidate mechanisms and predictors of age-related decline [14,18-22]. Gait speed is an integrative marker reflecting multisystem physiological function, including musculoskeletal, cardiorespiratory, neurological, and cognitive domains, and is widely recognized as a “sixth vital sign” for aging and frailty [23]. Muscle performance specifically quantifies neuromuscular capacity and is a direct indicator of sarcopenia and physical reserve [20,24].

Several studies have demonstrated that slower gait speed and reduced muscle performance are associated with recurrent falls. However, most prior research has focused on older and often more functionally impaired populations [22,25-27]. Studies including middle-aged adults have shown that these measures are associated with mobility difficulties [14,28], whereas a study of community-dwelling adults aged 50 to 65 years reported no association with adverse events, including falls [14]. Evidence specifically examining the relationship between physical performance and falls in middle-aged populations therefore remains limited. Examining this association in independent, community-dwelling, middle-aged adults may help identify risk factors that emerge before overt functional decline.

Using longitudinal MIDUS data, this study primarily examined whether midlife gait speed and muscle performance were associated with recurrent falls later in life. In addition, longitudinal changes in gait speed and muscle performance from midlife onward were examined to characterize their trajectories across adulthood. Secondary analyses assessed whether these same measures were associated with subjective age and self-rated health at follow-up.

## Methods

### Study Design and Participants

We conducted a longitudinal analysis using data from the MIDUS cohort, focusing on wave 2 (baseline, 2004-2009) [12] and wave 3 (follow-up, 2013-2021) [13]. Although MIDUS is a prospective cohort study, the present investigation represents a secondary analysis linking midlife physical performance at wave 2 with outcomes assessed at wave 3. This study is reported in accordance with the STROBE (Strengthening the Reporting of Observational Studies in Epidemiology) guidelines [29], and the completed checklist is available in Table S1 in Multimedia Appendix 1.

The analytic sample was defined based on eligibility at wave 2 and was restricted to relatively healthy, independent adults aged 45 to 64 years who did not use walking aids and were able to complete the 50-foot walking test. To minimize confounding and strengthen internal validity, participants with conditions likely to influence physical performance or fall risk were excluded. Specifically, participants were excluded if they reported a history of stroke or transient ischemic attack, head injury requiring an overnight emergency department or hospital stay, neurological disorder or cancer, blindness in one or both eyes, prior antiepileptic medication use, or opioid use at wave 2.

Physical performance measures (gait speed and muscle performance) obtained at wave 2 were examined in relation to the primary outcome of recurrent falls reported at wave 3. At wave 3, participants were asked to report the number of falls experienced during the previous 12 months. Responses were recorded as a count variable and subsequently categorized as recurrent falls (≥2 falls) versus nonrecurrent (0-1 falls). In addition, we examined changes in physical performance between the 2 waves. Secondary exploratory analyses examined associations between baseline physical performance and subjective age and self-rated health at follow-up. All MIDUS data are fully deidentified and anonymized, ensuring compliance with ethical research standards.

### Physical Performance Measures

As part of the MIDUS data collection, participants completed the 50-foot timed walk test twice. They were instructed to walk at their usual comfortable speed from a standing start to a designated turnaround point and back. For the present analysis, gait speed (m/s) was calculated by dividing the total distance (50 feet; 15.24 m) by completion time in seconds. The average of the 2 trials was used in all analyses.

During the MIDUS assessment, handgrip strength was measured using a handheld dynamometer and recorded in kilograms (equivalent to kilogram-force). Participants performed 3 maximal-effort trials. For the present analysis, the mean value across the 3 trials of the dominant hand was used.

Participants also completed the 5-repetition sit-to-stand test as part of the MIDUS protocol. For the present analysis, 2 indicators of lower-limb muscle performance were derived from this test: the total sit-to-stand completion time (seconds) and lower-extremity muscle power. Sit-to-stand completion time was obtained directly from the MIDUS dataset, whereas lower-extremity muscle power was calculated for the present analysis using an established allometric equation incorporating body mass, height, chair height (approximated as 45 cm), and total sit-to-stand duration [30]:



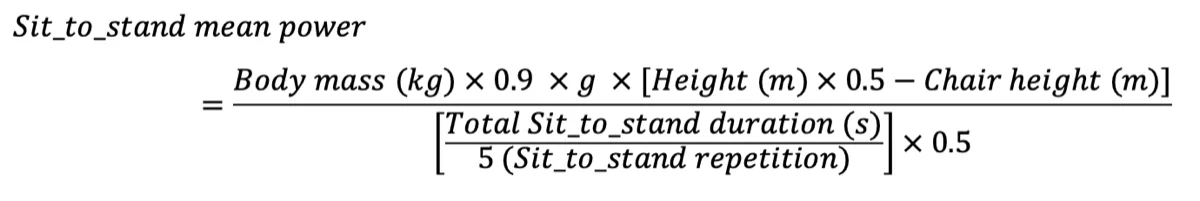



### Self-Perceived Age and Health

Subjective age was measured across MIDUS waves by asking, “Many people feel older or younger than they actually are. What age do you feel most of the time?” Participants reported the age they felt in years as an open-ended numeric response, which was treated as a continuous variable in subsequent analyses. Self-rated health was assessed using 2 ordinal items: general physical health (“In general, would you say your physical health is excellent, very good, good, fair, or poor?”; 1=excellent to 5=poor) and comparative health relative to age peers (“Compared to others your age, would you say your health is much better, somewhat better, about the same, somewhat worse, or much worse?”; 1=much better to 5=much worse).

### Statistical Analysis

Descriptive statistics are presented as mean (SD) or median with IQR, as appropriate. Continuous variables were assessed for distribution using visual inspection of histograms and Q-Q plots. Several complementary modeling approaches were used to address the study objectives. For the primary analysis, logistic regression examined whether midlife gait speed and muscle performance (grip strength, sit-to-stand completion time, and lower-limb muscle power) were associated with recurrent falls (≥2 falls in the preceding 12 months) at follow-up. Except for sit-to-stand completion time, all physical measures were directionally inverted prior to modeling so that odds ratios (ORs) reflected the effect of poorer performance. Age-related changes in gait speed and muscle performance between MIDUS waves were examined using linear mixed-effects models with random intercepts for participant ID and adjustment for baseline age and follow-up interval to estimate within-person change over time.

Secondary analyses evaluated associations of physical measures with subjective age and self-perceived health at follow-up, using linear regression for subjective age and ordinal regression for both self-perceived health measures (general physical health and comparative health). Primary and secondary regression models were adjusted for follow-up interval, baseline age, sex, BMI, height, baseline symptom burden (count of self-reported symptoms and chronic conditions), and the baseline value of the respective dependent variable.

Additionally, subgroup analyses were conducted by stratifying participants by age at follow-up (<65 or ≥65 years). The magnitude of associations in linear models was assessed using standardized regression coefficients (β), with values of 0.1, 0.3, and 0.5 denoting small, medium, and large effects, respectively [31,32]. For multiple testing, separate domains were defined in advance. Consistent with the literature, gait speed and muscle performance were treated as distinct domains. Gait speed is an integrative measure of multisystem physiological function [23], whereas grip strength, sit-to-stand time, and sit-to-stand power together index neuromuscular capacity [20,24]. The significance threshold was therefore adjusted within the muscle performance domain (*P*<.016) but not for gait speed, and unadjusted *P* values with 95% CIs are reported throughout.

As a sensitivity analysis, each primary adjusted and unadjusted model, for gait speed and each muscle performance measure, was refitted in participants free of recurrent falls at baseline (wave 2). This tested whether midlife performance was associated with newly emerging, rather than persistent, recurrent falls at follow-up. These sensitivity analyses were not adjusted for multiplicity and were interpreted as supportive rather than confirmatory. Statistical analyses were conducted in R (version 4.3.0; R Foundation for Statistical Computing) using the *tidyverse* and *lme4* packages [33-35].

### Ethical Considerations

This study is a secondary analysis of deidentified data from the MIDUS Biomarker Project. The original data collection received ethics approval from the institutional review board at the University of Wisconsin-Madison, and informed consent was obtained from all participants by the MIDUS investigators.

## Results

### Overview

The participant selection process and the final analytic sample are shown in Figure 1. The initial dataset included 459 middle-aged participants at baseline (MIDUS wave 2). After applying prespecified exclusion criteria and removing cases with missing data, the final analytic sample consisted of 309 participants, 45% (n=141) of whom were male. The mean age was 54.62 (SD 5.60) years at baseline and 66.26 (SD 5.66) years at follow-up. At follow-up, 181 (58.6%) participants were 65 years or older. The mean interval between waves was 11.64 (SD 1.25) years. The median baseline count of self-reported symptoms and chronic conditions was 3 (IQR 2-4). A total of 63 (20.4%) participants reported recurrent falls (≥2 falls) at follow-up; of these, 15 (23.8%) had already been recurrent fallers at wave 2, and 48 (76.2%) were newly recurrent at follow-up. Demographic characteristics, health indicators, and physical performance measures at MIDUS waves 2 and 3 are presented in Table 1.

**Figure 1:**
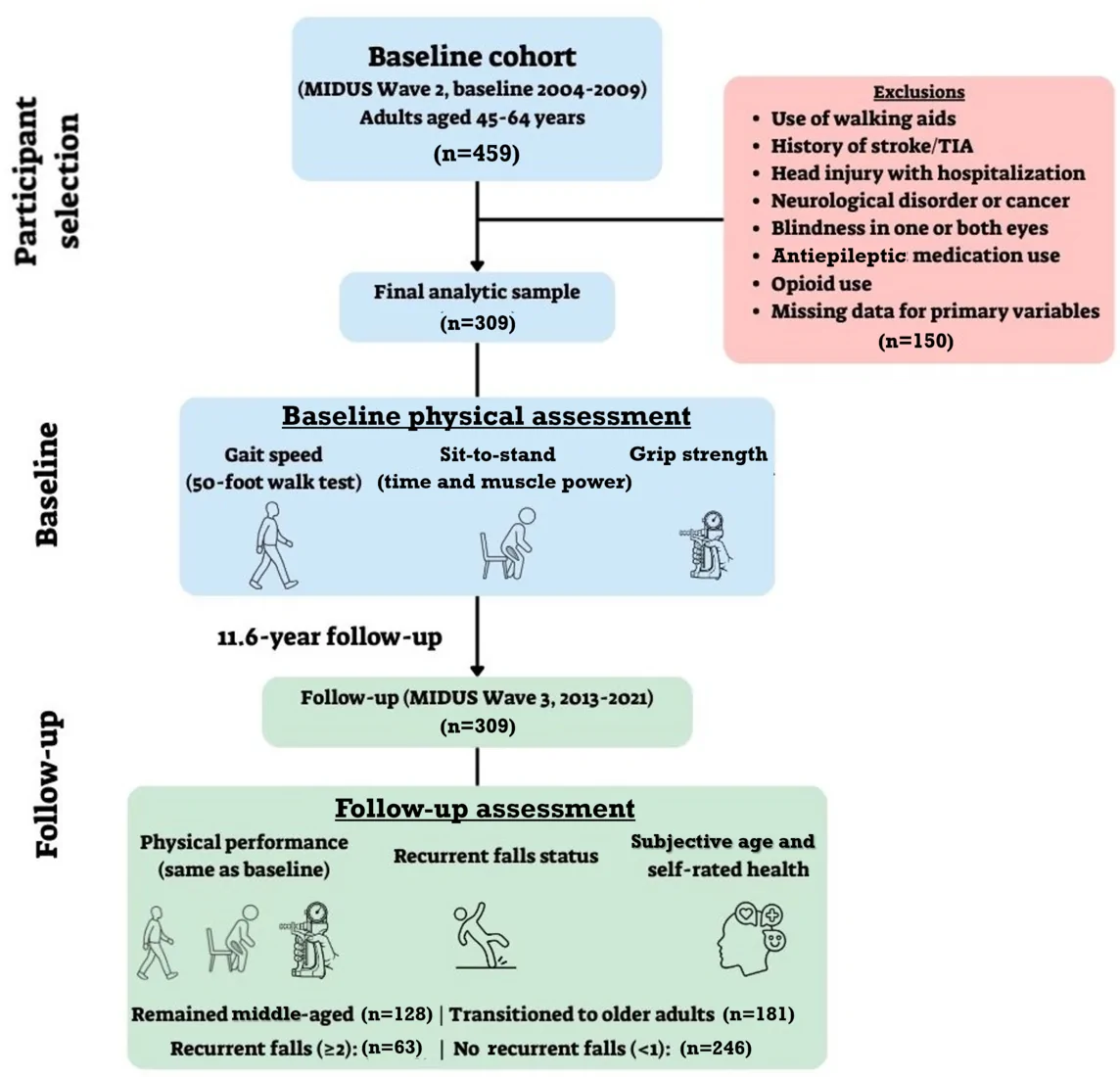
Flow diagram of participant selection and the analytic sample from the Midlife in the United States (MIDUS) cohort. TIA: transient ischemic attack.

**Table 1:** Sample characteristics in wave 2 and wave 3.

Variable		MIDUS wave 2 (baseline)	MIDUS wave 3 (follow-up)
**General characteristics (N=309)**			
	Age (y), mean (SD)^a^	54.62 (5.60)	66.26 (5.66)
	Female, n (%)	168 (54.4)	168 (54.4)
	BMI (kg/m^2^), mean (SD)	29.25 (5.85)	29.74 (6.43)
	Reported symptoms and chronic conditions, median (IQR)	3 (2-4)	4 (2-6)
	Recurrent fallers^b^, n (%)	55 (17.8)	63 (20.4)
**Physical performance, mean (SD)**			
	Gait speed^c^ (m/s)	1.11 (0.20)	1.11 (0.20)
	Grip strength (kg)	36.40 (12.15)	30.99 (10.56)^d^
	STS^e^ time (s)	9.24 (3.21)	10.52 (4.10)^d^
	STS mean power (W)	360.55 (163.47)	312.96 (141.26)^d^
**Self-perceived age and health (n=280)**			
	Subjective age (y), mean (SD)	42.28 (10.80)	49.57 (9.59)
	Differences between true age and subjective age, mean (SD)^f^	9.36 (9.42)	11.35 (8.40)
	Self-perceived general physical health, median (IQR)^g^	2 (2-3)	2 (2-3)
	Self-perceived health compared with age peers, median (IQR)	2 (1-3)	2 (1-2)

^a^Chronological age (n=309) and subjective age (n=280) are reported for different analytic samples.

^b^Recurrent falls were defined as the self-reported occurrence of 2 or more falls in the 12 months preceding each assessment.

^c^Gait speed was calculated from the 50-foot walk test as distance divided by completion time.

^d^*P*<.001 for changes in physical performance between waves, analyzed using linear mixed-effects models adjusted for baseline age and the time interval between assessments.

^e^STS: sit-to-stand.

^f^The age-difference values represent the mean within-person differences between chronological and subjective age calculated within the n=280 subsample and therefore do not equal the differences between the means reported above.

^g^Self-perceived general and comparative health were measured on a 1 to 5 scale, with higher scores indicating worse perceived health.

### Association of Physical Performance Measures at Wave 2 With Recurrent Falls at Wave 3

Among the physical performance measures examined, only gait speed was significantly associated with recurrent falls. Slower gait speed was associated with higher odds of 2 or more falls at follow-up in the total sample (OR 1.43, 95% CI 1.05-1.95; *P*=.02), corresponding to a 43% increase in the odds of recurrent falls per 1 SD decrease in gait speed. In the age-stratified adjusted models, the association was stronger among participants who were 65 years or older at follow-up (OR 1.72, 95% CI: 1.11-2.74; *P*=.02), whereas no significant association was observed among participants younger than 65 years at follow-up (OR 1.15, 95% CI 0.73-1.86; *P*=.55). Figure 2 illustrates the predicted probability of recurrent falls at follow-up according to baseline gait speed in the total sample and age-stratified analyses. In contrast to gait speed, no statistically significant associations were observed between sit-to-stand time, calculated sit-to-stand power, and grip strength and recurrent falls in the total sample or in the age-stratified analyses. Results from the unadjusted and adjusted models are presented in Table 2. In a sensitivity analysis restricted to the 254 participants who were not recurrent fallers at baseline, slower midlife gait speed remained significantly associated with recurrent falls in the total sample (adjusted OR 1.48, 95% CI 1.05-2.13; *P*=.03), and this association was stronger among participants 65 years or older (adjusted OR 1.89, 95% CI 1.16-3.17; *P*=.02). Grip strength also showed a stronger association with a narrower 95% CI, most pronounced among participants 65 years or older, where lower grip strength was significantly associated with becoming a new recurrent faller (adjusted OR 2.13, 95% CI 1.02-4.67; *P*=.049). This finding was not seen in the unadjusted model or the primary, nonrestricted analysis. However, this result did not meet the Bonferroni-corrected threshold (*P*<.016). Results of the sensitivity analysis are summarized in Table S2 in Multimedia Appendix 1.

**Figure 2:**
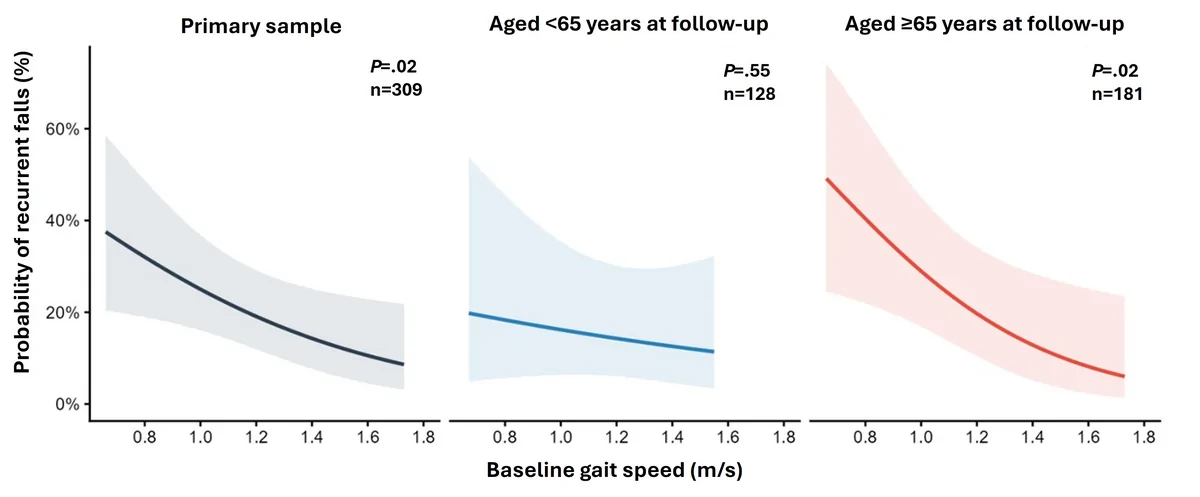
Probability of recurrent falls (≥2) by baseline gait speed, adjusted for baseline covariates measured at Midlife in the United States (MIDUS) wave 2, including age, sex, BMI, height, baseline fall count, aggregate count of self-reported medical diagnoses and chronic conditions, and the time interval (years) between MIDUS waves.

**Table 2:** Logistic regression models examining the association between baseline physical performance and recurrent falls at follow-up.

Independent variable^a^ and total sample/age category at follow-up		Unadjusted OR^b^ (95% CI)	*P* value	Adjusted OR (95% CI)^c^	*P* value
**Gait speed (m/s)^d^**					
	Total sample	1.35 (1.02-1.81)	.04	1.43 (1.05-1.95)	.02
	<65 y	1.20 (0.79-1.86)	.39	1.15 (0.73-1.86)	.55
	≥65 y	1.48 (1.01-2.21)	.047	1.72 (1.11-2.74)	.02
**Sit-to-stand** **time (s)**					
	Total sample	1.06 (0.80-1.39)	.67	1.10 (0.82-1.47)	.50
	<65 y	1.15 (0.75-1.71)	.50	1.05 (0.66-1.64)	.80
	≥65 y	0.99 (0.67-1.42)	.96	1.06 (0.69-1.60)	.76
**Power during sit-to-stand (W)^d^**					
	Total sample	1.03 (0.79-1.38)	.82	1.14 (0.78-1.69)	.52
	<65 y	0.89 (0.59-1.37)	.61	0.87 (0.50-1.51)	.62
	≥65 y	1.16 (0.79-1.76)	.45	1.39 (0.78-2.60)	.28
**Grip strength (kg)^d^**					
	Total sample	1.12 (0.85-1.49)	.43	1.34 (0.87-2.09)	.19
	<65 y	1.05 (0.69-1.61)	.83	1.04 (0.54-1.95)	.90
	≥65 y	1.19 (0.82-1.77)	.36	1.63 (0.86-3.19)	.14

^a^Age category represents the participant’s chronological age at the time of follow-up (MIDUS wave 3).

^b^OR: odds ratio.

^c^Adjusted models controlled for baseline covariates measured at MIDUS wave 2, including age, sex, BMI, height, baseline fall count, aggregate count of self-reported medical diagnoses and chronic conditions, and the time interval (years) between MIDUS waves.

^d^For all physical performance measures except sit-to-stand time, variables were coded so that higher odds ratio indicate poorer performance.

### Longitudinal Changes in Gait Speed and Muscle Performance

Gait speed did not show a statistically significant change between MIDUS waves, remaining stable at 1.11 (SD 0.20) m/s at both baseline and follow-up. In contrast, all muscle performance indicators declined over time. Grip strength showed the largest standardized decline, decreasing from 36.40 (SD 12.15) kg at baseline to 30.99 (SD 10.56) kg at follow-up, corresponding to an approximately 15% reduction (β=−0.47, 95% CI −0.53 to −0.40; *P*<.001). Sit-to-stand time increased from 9.24 (SD 3.21) seconds to 10.52 (SD 4.10) seconds, indicating slower task completion and worsening performance (β=0.34, 95% CI 0.21-0.48; *P*<.001). Sit-to-stand power decreased from 360.55 (SD 163.47) W to 312.96 (SD 141.26) W, indicating reduced lower-extremity power (β=−0.31, 95% CI −0.41 to −0.21; *P*<.001). Together, these findings indicate that muscle performance measures were more sensitive to age-related change across the follow-up period than gait speed.

### Association of Physical Performance Measures at Wave 2 With Subjective Age and Self-Rated Health at Wave 3

This analysis included 280 participants. In unadjusted models, slower gait speed was associated with poorer self-rated health on both follow-up health measures. Each SD decrease in gait speed was associated with 32% higher odds of poorer self-perceived physical health in the total sample (OR 1.32, 95% CI 1.05-1.64; *P*=.02) and 39% higher odds among participants younger than 65 years at follow-up (OR 1.39, 95% CI 1.06-1.82; *P*=.01). A similar pattern was observed for health compared with age peers in the total sample (OR 1.49, 95% CI 1.19-1.89; *P*<.001) and among participants younger than 65 years (OR 1.56, 95% CI 1.19-2.08; *P*=.001), whereas no significant associations were observed among participants aged 65 years or older. However, after adjustment for baseline covariates, these associations were no longer statistically significant.

Gait speed was not associated with subjective age in unadjusted analyses (β=−0.03, 95% CI −1.34 to 0.93; *P*=.59). However, in fully adjusted models, faster gait speed was independently associated with a younger subjective age at follow-up, corresponding to an approximately 1-year younger subjective age for every 0.20 m/s increase in gait speed (β=−0.10, 95% CI −0.20 to −0.006; *P*=.04). This association did not reach statistical significance in either age-stratified subgroup.

No significant associations were observed between the other physical performance measures and either self-rated health or subjective age in the total sample or age-stratified analyses.

## Discussion

### Principal Findings

This longitudinal analysis of the MIDUS cohort examined whether midlife gait speed and muscle performance were associated with recurrent falls later in life. Slower midlife gait speed was associated with a 43% increase in the odds of recurrent falls at follow-up, whereas none of the muscle performance measures showed a significant association with future falls.

The association between gait speed and recurrent falls emerged despite the cohort maintaining high functional status, as indicated by a stable mean walking speed between waves (1.11, SD 0.20 m/s) that remained, on average, above commonly referenced geriatric risk thresholds (0.8-1.0 m/s) [4,26,27], and despite adjustment for fall history at baseline. Other longitudinal cohorts have similarly reported relative stability of gait speed across midlife and early older adulthood, consistent with our findings [36,37]. Although this average gait speed suggests preserved mobility at the group level, it may obscure meaningful between-individual differences in walking performance. Thus, the observed association with future falls likely reflects differences already present during midlife rather than progressive age-related decline, supporting the potential role of slower midlife gait speed as an early marker of future fall vulnerability.

Prior research also indicates that gait speed reflects broader biological and functional processes already present in midlife. For example, Rasmussen et al [19] reported that slower gait speed at age 45 was associated with poorer physical and neurocognitive function. Similarly, findings from the Baltimore Longitudinal Study of Aging demonstrated that multiple motor impairments are linked to slow gait speed in mid-to-late life [28].

Evidence from longitudinal studies in midlife, however, remains limited and somewhat inconsistent. In a cohort of community-dwelling adults aged 50 to 65 years, Segaux et al [14] reported that gait speed was not associated with subsequent adverse events, including nonaccidental falls. Several methodological differences may explain this discrepancy. First, participants in the study by Segaux et al [14] had exceptionally high functional status, with a median gait speed of 1.43 m/s and very few participants meeting criteria for slowness (<1 m/s). Second, the follow-up period in that study was considerably shorter than in the present analysis, which followed participants for more than a decade. Finally, differences in gait assessment may also contribute, as the MIDUS 50-foot walk includes a turning phase that places greater demands on dynamic balance than the straight-path walking tests [38,39] used in the Segaux et al [14] study. Together, these differences may explain the contrasting findings and highlight the need for further longitudinal research focusing on midlife populations to clarify the role of gait speed in predicting later fall risk. It would also be informative for future studies to examine midlife populations stratified into early and late midlife groups.

Consistent with previous studies reporting differences between younger and older middle-aged adults [40,41], our age-stratified analyses showed that the association between midlife gait speed and recurrent falls was primarily evident among participants who were 65 years or older at follow-up, whereas no statistically significant association was observed among those younger than 65 years. Nevertheless, these findings should be interpreted cautiously and require confirmation in larger longitudinal cohorts.

In contrast to gait speed, muscle performance indices, including sit-to-stand time, lower-extremity power, and grip strength, are considered particularly sensitive to age-related changes during midlife [24,30,37,42,43], and declined significantly over follow-up. However, none of these measures were associated with recurrent falls in the present cohort. Falls in community-dwelling adults commonly occur during walking [44], a complex task that requires continuous dynamic balance supported by sensorimotor integration and cognitive processing [19,45]; thus, gait speed likely reflects the integrity of multiple physiological systems involved in maintaining stability. In comparison, isolated aspects of muscular capacity assessed under relatively controlled conditions may be less sensitive to the complex demands underlying walking stability, particularly during midlife when substantial muscle deconditioning is often absent [46].

Unlike our findings, the China Health and Retirement Longitudinal Study (CHARLS) reported that weak grip strength was associated with increased fall risk over a 4-year follow-up period that included middle-aged adults [22]. However, the CHARLS cohort included participants with conditions such as stroke, rheumatoid arthritis, and kidney disease that independently increase fall risk [22], whereas the present study excluded participants with major neurological and mobility-impairing conditions. In addition, mean midlife grip strength in our cohort was 45 kg for men and 28 kg for women, whereas CHARLS identified weaker grip strength (<30 kg for men and <20 kg for women) as a fall risk factor [22]. These differences in participant characteristics and grip strength distribution likely contributed to the discrepant findings. Therefore, our findings suggest that slower midlife gait speed may reflect early vulnerability to future fall risk, even when conventional measures of muscle performance remain within normal ranges. These findings underscore the need for further longitudinal studies to identify indicators of fall risk before overt functional decline.

A secondary exploratory aim of the present investigation was to examine whether midlife physical performance was associated with subjective age and self-rated health later in life. Faster midlife gait speed was associated with younger subjective age at follow-up after accounting for baseline participant characteristics; however, the estimated magnitude was small, corresponding to approximately a 1-year younger subjective age for a 0.20 m/s increase in gait speed. Associations between gait speed and self-perceived health were observed only before baseline characteristics were accounted for. Although these secondary findings should be interpreted as complementary to the primary fall-related outcomes, they are in line with previous research showing that gait speed correlates with self-reported physical limitations, lower self-rated health, and poorer scores on health-related quality of life instruments such as the 36-Item Short Form Survey [19,47]. Together, these findings suggest that walking performance may relate not only to future fall risk but also to subjective perceptions of health and aging.

Notably, participants in both MIDUS waves generally reported feeling younger than their chronological age, with a mean subjective age approximately 10 years lower, a pattern often interpreted as reflecting psychological resilience [48]. This pattern may also reflect the relatively healthy composition of the analytic sample. Associations between objective gait performance and perceived aging have rarely been examined in such healthy midlife populations, making this a potentially novel observation that warrants further investigation. To obtain a more comprehensive understanding of subjective and objective functioning during midlife, future studies should examine the relationship between objective physical performance and midlife-specific self-perception tools, such as the 16-item Mobility in Middle-Age Questionnaire [49].

### Limitations

Several limitations should be considered when interpreting our findings. First, the primary gait speed association should be interpreted in the context of the multiplicity approach used in this study. Although gait speed and muscle performance were defined in advance as separate domains, if a single Bonferroni correction had been applied across all 4 physical performance measures (*P* <.0125), the association between gait speed and recurrent falls would not have met that threshold. Second, the observational design and reliance on self-reported falls and health conditions may introduce recall bias. Third, although exclusion criteria were applied to restrict the analytic sample to generally independent participants and strengthen internal validity, they also limit generalizability to the broader middle-aged population and reduce the sample size and number of recurrent fall events, particularly in analyses stratified by age category. Because MIDUS is an established closed cohort and the sample size was fixed by the number of eligible participants with complete data at both waves, no a priori power calculation was applicable. Although participants with major neurological conditions were excluded, residual confounding from unreported or undiagnosed health conditions cannot be ruled out and may have influenced performance on the physical tests. In addition, sit-to-stand muscle power was estimated using a validated equation rather than measured directly. Although this approach is established [30], the assumption of a standard chair height may have reduced individual-level precision and potentially obscured subtle associations. Finally, baseline subjective measures were collected during the MIDUS telephone interview, which preceded the biomarker assessment by approximately 2 to 3 years. This temporal gap may have introduced some measurement misalignment between subjective and objective variables, potentially affecting the precision of model adjustment. Despite these limitations, the study benefits from a longitudinal design with over a decade of follow-up and the use of objective physical performance measures within a well-characterized population-based cohort, providing a valuable opportunity to examine early functional markers of fall risk beginning in midlife.

### Conclusions

Using data from the MIDUS cohort, this study provides evidence that vulnerability to recurrent falls may be detectable as early as midlife. Slower self-selected walking speed was associated with higher odds of recurrent falls at follow-up, despite stable average gait speed over time. In contrast, muscle performance indices declined with age but were not significantly associated with recurrent falls in the primary analysis. Further longitudinal studies in larger midlife cohorts are warranted to confirm these findings.

## Appendix

Multimedia Appendix 1 STROBE checklist and sensitivity analysis results.

## Abbreviations

CHARLS: China Health and Retirement Longitudinal Study
MIDUS: Midlife in the United States
OR: odds ratio
STROBE: Strengthening the Reporting of Observational Studies in Epidemiology

## Data Availability

The data used in this study are publicly available from the Midlife in the United States (MIDUS) study via the Inter-university Consortium for Political and Social Research (ICPSR) repository [50].
