# Supplementary material for: Associations of Midlife Gait Speed and Muscle Performance With Falls in Later Life: Secondary Analysis of the MIDUS Cohort
Source: JMIR Aging. 2026 Oct 6;9:e103917. doi: 10.2196/103917 (PMC13684120; doi:10.2196/103917)
Supplement: Supplementary file 1 [file aging_v9i1e103917_app1.pdf]

Supplementary Table S1. STROBE Statement Checklist.
Study Title: Associations of Midlife Gait Speed and Muscle Performance with Falls in Later Life
Design: Observational Cohort Study (Secondary Analysis of the MIDUS Cohort)

Item No	Recommendation	Reported Location & Source Context
**Title and Abstract**		
1	(a) Indicate the study's design with a commonly used term in the title or the abstract.	*(a) Abstract ("Methods") explicitly states: "A retrospective longitudinal analysis used data from the Midlife in the United States (MIDUS) cohort."*
	(b) Provide in the abstract an informative and balanced summary of what was done and what was found.	*(b) Abstract provides a complete summary of Background, Objective, Methods, Results and Conclusions.*
**Introduction**		
2	Background/rationale: Explain the scientific background and rationale for the investigation being reported.	*Introduction, Paragraphs 1-4: Outlines the burden of falls, highlights midlife as a critical prevention window, defines the distinct system indicators of gait speed and muscle performance, and explains the knowledge gap in healthy middle-aged populations.*
3	Objectives: State specific objectives, including any prespecified hypotheses.	*Introduction, Paragraph 5: Explicitly states the primary objective (examine if midlife gait speed and muscle performance relate to future recurrent falls) and secondary exploratory objectives (subjective age and self-rated health).*
**Methods**		
4	Study design: Present key elements of study design early in the paper.	*Methods ("Study Design and Participants"): Described as a longitudinal analysis linking Wave 2 baseline measures (2004-2009) to Wave 3 follow-up outcomes (2013-2021).*
5	Setting: Describe the setting, locations, and relevant dates, including periods of recruitment, exposure, follow-up, and data collection.	*Methods ("Study Design and Participants"): Identifies the Midlife in the United States (MIDUS) cohort framework, listing Wave 2 (2004-2009) and Wave 3 (2013-2021).*
6	Participants:<br>(a) Give the eligibility criteria, and the sources and methods of selection of participants. Describe methods of follow-up.	*(a) Methods ("Study Design and Participants"): Detailed inclusion criteria (independent, community-dwelling, aged 45-64 years, completed 50-foot walk without aids) and comprehensive medical/neurological exclusion criteria.*

	(b) For matched studies, give matching criteria and number of exposed and unexposed.	*(b) Not applicable (unmatched cohort study).*
**7**	Variables: Clearly define all outcomes, exposures, predictors, potential confounders, and effect modifiers. Give diagnostic criteria, if applicable.	*Methods ("Physical performance measures", "Self-Perceived Age and Health", and "Statistical Analysis"): Defines exposure variables (gait speed, grip strength, sit-to-stand time, lower-limb power), primary outcome (recurrent falls: ≥2 falls in the preceding 12 months), and covariates adjusted (age, sex, BMI, height, baseline fall count, symptom burden, wave interval).*
**8**	Data sources/measurement: For each variable of interest, give sources of data and details of methods of assessment (measurement). Describe comparability of assessment methods if there is more than one group.	*Methods ("Physical performance measures" & "Self-Perceived Age and Health"): Describes the standardized MIDUS tools used.*
**9**	Bias: Describe any efforts to address potential sources of bias.	*Methods ("Study Design and Participants"): exclusion criteria minimized confounding; baseline fall count adjusted for reverse causation. Discussion (Limitations): notes possible recall and survivorship bias.*
**10**	Study size: Explain how the study size was arrived at.	*Results, Paragraph 1 & Figure 1: The study size was determined by the available subpopulation within the MIDUS cohort meeting all specific eligibility criteria (n = 309).*
**11**	Quantitative variables: Explain how quantitative variables were handled in the analyses. If applicable, describe which groupings were chosen and why.	*Methods ("Statistical Analysis"): Physical performance indicators were kept continuous and inverted (except STS time) so that ORs reflect poorer performance. Subgroup analyses were stratified using chronological age at follow-up (<65 vs. ≥65 years).*
**12**	Statistical methods:<br>(a) Describe all statistical methods, including those used to control for confounding.<br>(b) Describe methods used to examine subgroups and interactions.<br>(c) Explain how missing data were addressed.<br>(d) If applicable, explain how loss to follow-up was addressed.<br>(e) Describe any sensitivity analyses.	*(a) Methods ("Statistical Analysis"): Logistic regression for primary outcome, adjusting for baseline covariates. Mixed-effects models for longitudinal changes.*<br>*(b) Stratified regression models across age subgroups (<65 and ≥65 years).*<br>*(c) Complete-case analysis was used after removing missing variables (detailed in Figure 1).*<br>*(d) Figure 1 and Results, first paragraph: the analytic sample was restricted to participants with available data at both waves; no separate loss-to-follow-up analysis was performed*<br>*(e) Sensitivity analyses excluding participants with recurrent falls at baseline are described in the Statistical Analysis section and reported in the Results and Supplementary Table S1.*

**Results**		
**13**	Participants:<br>(a) Report numbers of individuals at each stage of study (e.g. numbers potentially eligible, examined for eligibility, confirmed eligible, included in the study, completing follow-up, and analysed.<br>(b) Give reasons for non-participation at each stage.<br>(c) Consider use of a flow diagram.	*(a, b, c) Fully detailed in Results (Paragraph 1) and visually systematically presented in Figure 1.*
**14**	Descriptive data:<br>(a) Give characteristics of study participants and information on exposures and potential confounders.<br>(b) Indicate number of participants with missing data for each variable of interest.<br>(c) Summarize follow-up time (e.g., average and total amount).	*(a) Comprehensive participant characteristics detailed in Results, Paragraph 1, and Table 1.*<br>*(b) Addressed via complete-case exclusion mapping in Figure 1.*<br>*(c) Results (Paragraph 1) and Table 1 report a mean follow-up interval of 11.64 ± 1.25 years.*
**15**	Outcome data: Cohort Study-Report numbers of outcome events or summary measures over time.	*Results Paragraph 1, and Table 1.*
**16**	Main results:<br>(a) Give unadjusted estimates and, if applicable, confounder-adjusted estimates and their precision (e.g., 95% confidence interval). Make clear which confounders were adjusted for and why they were included.<br>(b) Report category boundaries when continuous variables were categorized.<br>(c) If relevant, consider translating estimates of relative risk into absolute risk for a meaningful time period.	*(a, b) Results ("Association of physical performance measures at Wave 2 with recurrent falls at Wave 3") and also detailed in Table 2.*<br>*(c) Outlined via the predicted probability curves presented in Figure 2.*
**17**	Other analyses: Report other analyses done - e.g. analyses of subgroups and interactions, and sensitivity analyses.	*Results ("Association of physical performance measures... with subjective age and self-rated health"): Reports linear and ordinal regression results for secondary exploratory outcomes. Subgroup models by age category are presented throughout text and figures.*
**Discussion**		
**18**	Key results: Summarise key results with reference to study objectives.	*Discussion, Paragraph 1: Provides a clear, direct summary of the key findings matching the study objectives.*
**19**	Limitations: Discuss limitations of the study, taking into account sources of potential bias or imprecision. Discuss both direction and magnitude of any potential bias.	*Discussion, limitation paragraph.*

**20**	Interpretation: Give a cautious overall interpretation of results considering objectives, limitations, multiplicity of analyses, results from similar studies, and other relevant evidence.	*Discussion section.*
**21**	Generalizability: Discuss the generalizability (external validity) of the study results.	*Discussion, Paragraph 7: Formally addresses how the application of stringent clinical criteria isolates internal mechanisms but simultaneously limits direct external validity to broader, less-healthy middle-aged populations.*
**Other Information**		
**22**	Funding: Give the source of funding and the role of the funders for the present study and, if applicable, for the original study on which the present article is based.	*Declarations ("Funding"): Explicitly states: "This research did not receive funding."*

Supplementary Table S2. Logistic regression models examining the association between baseline physical performance and recurrent falls at follow-up: sensitivity analysis excluding baseline recurrent fallers.

Measure	Age category at MIDUS 3	Model	OR	95% CI low	95% CI high	p-value
Gait speed	All	Unadjusted, sensitivity subsample	1.43	1.03	2.00	**0.033**
Gait speed	All	Adjusted, sensitivity subsample	1.48	1.05	2.13	**0.027**
Gait speed	<65 years	Unadjusted, sensitivity subsample	1.18	0.72	1.96	0.532
Gait speed	<65 years	Adjusted, sensitivity subsample	1.16	0.67	2.08	0.608
Gait speed	≥65 years	Unadjusted, sensitivity subsample	1.61	1.05	2.56	**0.031**
Gait speed	≥65 years	Adjusted, sensitivity subsample	1.89	1.16	3.17	**0.012**
Grip strength	All	Unadjusted, sensitivity subsample	1.08	0.79	1.49	0.639
Grip strength	All	Adjusted, sensitivity subsample	1.30	0.79	2.13	0.303
Grip strength	<65 years	Unadjusted, sensitivity subsample	0.94	0.58	1.54	0.815
Grip strength	<65 years	Adjusted, sensitivity subsample	0.81	0.36	1.69	0.595
Grip strength	≥65 years	Unadjusted, sensitivity subsample	1.19	0.79	1.85	0.405
Grip strength	≥65 years	Adjusted, sensitivity subsample	2.13	1.02	4.67	**0.049**
STS time	All	Unadjusted, sensitivity subsample	1.12	0.82	1.52	0.453
STS time	All	Adjusted, sensitivity subsample	1.17	0.84	1.62	0.332
STS time	<65 years	Unadjusted, sensitivity subsample	1.16	0.71	1.83	0.539
STS time	<65 years	Adjusted, sensitivity subsample	1.16	0.67	1.94	0.586

STS time	≥65 years	Unadjusted, sensitivity subsample	1.10	0.72	1.65	0.650
STS time	≥65 years	Adjusted, sensitivity subsample	1.23	0.78	1.94	0.371
STS power	All	Unadjusted, sensitivity subsample	1.03	0.76	1.45	0.842
STS power	All	Adjusted, sensitivity subsample	1.20	0.78	1.89	0.411
STS power	<65 years	Unadjusted, sensitivity subsample	0.89	0.56	1.47	0.644
STS power	<65 years	Adjusted, sensitivity subsample	0.90	0.47	1.72	0.744
STS power	≥65 years	Unadjusted, sensitivity subsample	1.15	0.76	1.82	0.530
STS power	≥65 years	Adjusted, sensitivity subsample	1.59	0.85	3.23	0.170

STS = sit-to-stand, OR = odds ratio per 1-SD change in the predictor; CI = confidence interval. Bold rows indicate $p < 0.05$ (Bonferroni correction threshold for muscle performance measures $p < 0.016$). Both unadjusted (predictor alone) and adjusted (covariate-adjusted) models are reported. Adjusted models controlled for BMI, height, sex, age, symptom burden, and years between clinical visits.
